# Rhabdomyosarcoma masquerading as lymphadenopathy in a patient with newly diagnosed Hodgkin’s lymphoma

**DOI:** 10.1186/s12957-016-0846-0

**Published:** 2016-04-02

**Authors:** Joseph Dergan, Sandeep Sirsi, Armand Asarian, Elizabeth Guevara, Philip Xiao

**Affiliations:** Department of Surgery, The Brooklyn Hospital Center, Icahn School of Medicine at Mount Sinai, Brooklyn, NY 11201 USA; Department of Hematology/Oncology, The Brooklyn Hospital Center, Icahn School of Medicine at Mount Sinai, Brooklyn, NY 11201 USA; Department of Pathology and Laboratory Medicine, The Brooklyn Hospital Center, Icahn School of Medicine at Mount Sinai, Brooklyn, NY 11201 USA

**Keywords:** Hodgkin’s lymphoma, Nodular sclerosing type, Lymphadenopathic rhabdomyosarcoma, Alveolar type

## Abstract

**Background:**

Hodgkin’s lymphoma (HL) is a rare malignancy which often presents with lymphadenopathy and classic “B symptoms” of weight loss, fever, and night sweats. Additional masses or nodes could easily be presumed to be a result of the initial diagnosis. On the other hand, adult rhabdomyosarcoma is a rare malignancy presenting with a new mass in a patient with previous diagnosis of Hodgkin’s lymphoma. In both cases, a tissue diagnosis should be obtained to appropriately confirm the diagnosis.

**Case presentation:**

We present a case of a 64-year-old male who presents with right axillary lymphadenopathy, diagnosed as Hodgkin’s lymphoma. He subsequently developed left inguinal lymphadenopathy without the classic B symptoms of HL. Excisional biopsy revealed rhabdomyosarcoma. Stage III Hodgkin’s lymphoma (lymph node involvement on both sides of the diaphragm) is not commonly seen without typical B symptoms. Once the diagnosis of two primary malignancies is made, the dilemma becomes determining the treatment course. In the case of Hodgkin’s lymphoma and rhabdomyosarcoma, there is some overlap in the chemotherapeutic regimen and use of radiation.

**Conclusions:**

This case illustrates the importance of careful examination of Hodgkin’s lymphoma patients and consideration of additional tissue diagnoses in atypical presentations of new masses or lymphadenopathy on the opposite side of the diaphragm.

## Background

Hodgkin’s lymphoma (HL) is an uncommon malignancy of the lymphatic system, with an incidence of 2.5 per 100,000 [1]. It is well known to have a favorable prognosis, with a 5-year survival greater than 85 % and is thus viewed as a potentially curable disease. There are five histologic types currently described: nodular sclerosing (most common), mixed cellularity, lymphocyte rich (best prognosis), lymphocyte depleted (worst prognosis), and nodular lymphocyte predominant. With the exception of nodular lymphocyte predominant, all of these are characterized by the presence of Reed-Sternberg (RS) cells (typically B lymphocytes with CD 15 and CD 30 antigen positivity). Nodular sclerosing type, which has a strong genetic component, commonly involves the mediastinum and has CD20 antigen positivity.

Rhabdomyosarcoma (RMS) is a malignant tumor of striated muscle similar to other high-grade soft tissue sarcomas. It is the most common soft tissue sarcoma in children (90 % of cases are diagnosed under 25 years of age; 60–70 % of these are diagnosed under 10 years of age) but a rare diagnosis in adults, with only an approximated 400 new cases in the USA each year [2]. Even more rare is the diagnosis of “lymphadenopathic” RMS, with only four prior cases described in the literature [3–5]. Overall 5-year survival is poor compared to that of HL, at 40 % [6]. Head and neck is the most common anatomic location for these tumors. Five histologic types are described: embryonal (most common), alveolar (worst prognosis; commonly found in the extremities, trunk, perianal, and perirectal areas), botryoid embryonal, spindle cell embryonal, and anaplastic/undifferentiated [7]. Here, we report a case of lymphadenopathic rhabdomyosarcoma discovered in a patient with concurrent diagnosis of Hodgkin’s lymphoma.

## Case presentation

**Fig. 1:**
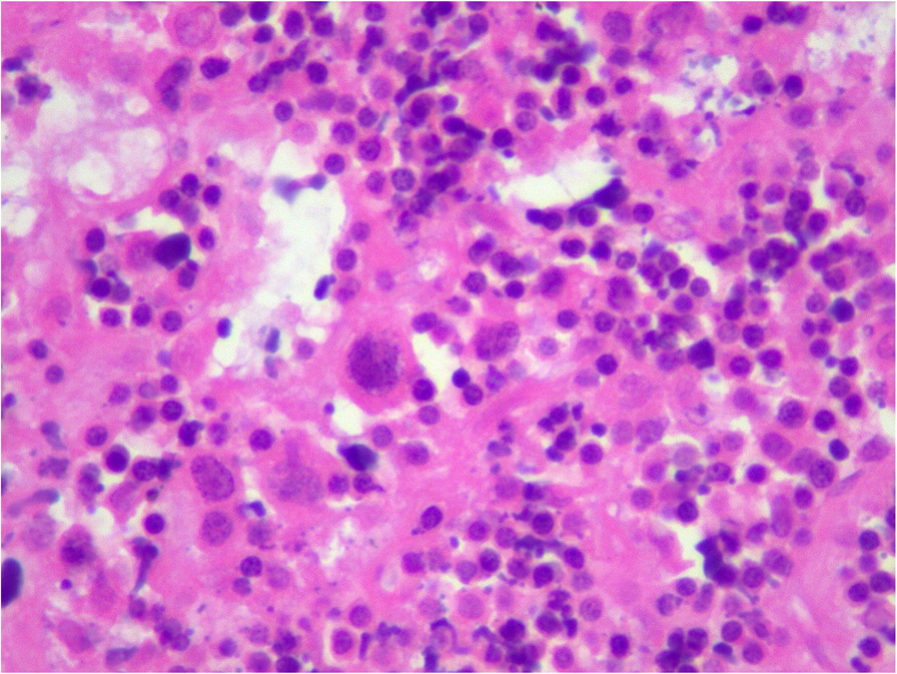
Microscopic examination reveals scattered Reed-Sternberg (RS) or mummified cells mixed with reactive inflammatory cells within fibrous nodular background (HE ×40)

**Fig. 2:**
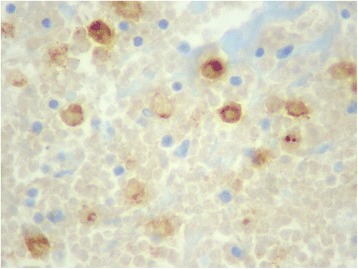
Immunohistochemical stain result reveals that RS cells are positive for CD30 (immunoperoxidase stain ×20)

A 64-year-old Hispanic male presented to an outpatient surgical setting at an inner-city community hospital complaining of a newly discovered right axillary mass. The patient had no past medical or surgical history other than a left inguinal hernia repair performed 6 years prior for a reducible, asymptomatic, left inguinal hernia. On physical examination, the patient was noted to have a large, firm, irregular, fixed, non tender mass in the right axilla, consistent with a pathologic lymph node. He denied any constitutional or B symptoms. He also denied any weight loss, sick contacts, or recent travel. The patient was offered a short course of oral antibiotic therapy and was asked to return to the office in 2 weeks. When the patient returned, the mass had slightly increased in size. The decision was made to perform a right axillary lymph node excisional biopsy. Microscopic examination revealed scattered Reed-Sternberg (RS) or mummified cells mixed with reactive inflammatory cells within fibrous nodular background (Fig. 1). Immunohistochemical stain results revealed that RS cells were positive for CD15 and CD30 (Fig. 2). The final pathology was reported as Hodgkin’s lymphoma, nodular sclerosing type.

He was immediately referred to a hematologist/oncologist, and the patient was prepared to initiate chemotherapy. Various tumor markers, hepatitis panel, and HIV testing were all negative. During this time, the patient developed right epitrochlear lymphadenopathy discovered by the oncologist. When seen again by the surgeon during a postoperative visit, the patient complained of left scrotal pain with an associated left inguinal mass, which he attributed to a recurrence of his previously repaired left inguinal hernia. Upon close examination, it was determined that the patient had left inguinal lymphadenopathy, with characteristics similar to the right axillary contents that were previously excised. The oncologist, once notified of this finding, requested a PET/CT scan. The scan revealed multiple enlarged right subclavian, right axillary, and left inguinal lymph nodes with moderate to significant FDG uptake. Based on the nature of Hodgkin’s lymphoma, the recommendation was made for an excisional biopsy of the left inguinal lymph node due to the low likelihood of the mass having the same HL origin (as would be more likely with non-Hodgkin’s lymphoma).

**Fig. 3:**
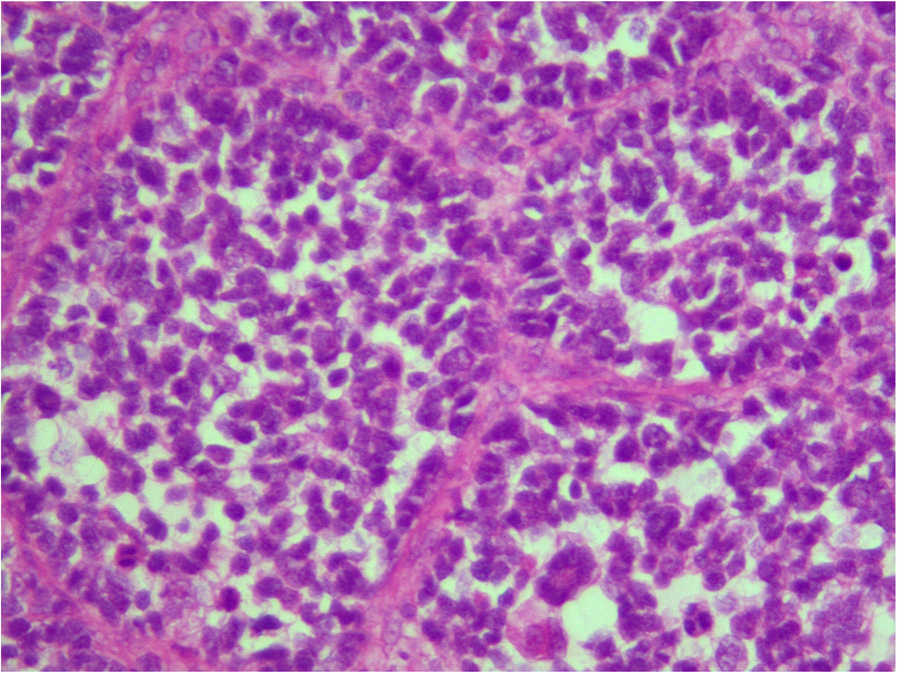
Microscopic examination reveals thin fibrous septae surrounded by small round blue cells with an alveolar growth pattern (HE ×20)

A left inguinal lymph node excisional biopsy was performed concurrently with placement of a left subclavian Mediport. Grossly, the excised mass had a similar appearance to the right axillary lymph nodes removed 2 months earlier. It was an encapsulated, flesh colored structure. Unexpectedly, the microscopic examination revealed thin fibrous septae surrounded by small round blue cells with an alveolar growth pattern. Immunohistochemical and molecular studies confirmed the diagnosis of rhabdomyosarcoma, alveolar type (Fig. 3). The pathologic slides were sent to a tertiary center and reviewed for a second opinion, and the diagnosis was concurred. The dilemma now faced is this: with two primary neoplasms, which one should be treated first and what modalities of treatment would be most effective? Given that this presentation is exceedingly rare, the patient was referred to a highly specialized tertiary cancer center in order to provide the best multidisciplinary approach to this oncologic dilemma.

### Discussion

Hodgkin’s lymphoma typically presents as painless lymphadenopathy. The so called B symptoms, which include weight loss, fever, and night sweats, may be present and implicate a poorer prognosis. Due to mediastinal involvement, the nodular sclerosing type may present with chest pain, cough, and shortness of breath. Often, a plethora of diagnostic modalities aid in the diagnosis of Hodgkin’s lymphoma, but ultimately, a tissue diagnosis is needed. This is usually obtained surgically with an excisional lymph node biopsy, as intact morphology is required for an accurate diagnosis. The one exception to this is in head and neck masses, where a fine needle aspiration (FNA) may be pursued in order to rule out squamous cell carcinoma, which is very common in this anatomic location. Once the diagnosis is made, a clinical stage is deciphered, in order to orchestrate the treatment plan.

Hodgkin’s lymphoma can be described in four stages as follows: stage I (a single lymph node area or single extranodal site), stage II (two or more lymph node areas on the same side of the diaphragm), stage III (lymph node areas on both sides of the diaphragm), and stage IV (disseminated or multiple involvement of the extranodal organs). Four treatments options are available depending on the nature of the disease: radiation therapy, induction chemotherapy, salvage chemotherapy, and hematopoietic stem cell transplantation. An example of an induction chemotherapeutic regimen is cyclophosphamide, doxorubicin, vincristine, and prednisone (CHOP) [8]. When induction chemotherapy fails, or the patient experiences relapse, a salvage chemotherapeutic regimen such as ifosfamide, carboplatin, and etoposide (ICE) can be initiated. Of note, HIV testing is obtained in all HL patients, as HAART therapy significantly improves outcomes in HIV positive patients with Hodgkin’s lymphoma [7].

Rhabdomyosarcoma typically presents as a mass or area of localized swelling, and less than half of these patients present with pain. However, symptoms can be specific to the anatomic location of the lesion (e.g., cranial nerve compression in head/neck tumors invading the skull base). Diagnosis is made with a surgical biopsy (usually an incisional biopsy). CT and MRI are helpful for planning surgical resection, as well as planning routes and doses for potential radiation therapy post operatively. Locoregional and metastatic disease can be investigated with a bone marrow biopsy, chest CT scan, technetium diphosphonate bone scan, and even a lumbar puncture (for parameningeal primaries). Mainstay therapy for rhabdomyosarcoma is surgical resection. High-risk histology and advanced stage may warrant chemotherapy with or without radiation. For example, non-metastatic alveolar rhabdomyosarcoma is usually treated with the vincristine, dactinomycin, and cyclophosphamide (VAC) protocol with the addition of radiation therapy [9].

Specifically relevant to this case, our patient initially presented with two groups of enlarged lymph nodes on the same side of the diaphragm and did not present with B symptoms. He subsequently developed involvement of a new group of lymph nodes on the other side of the diaphragm (left inguinal) without development of B symptoms. This was a rare presentation which raised the suspicion of the oncologist, prompting a second excisional biopsy. Specific to this case of Hodgkin’s lymphoma with synchronous rhabdomyosarcoma (alveolar type), overlapping treatment is available, thus making treatment options less cumbersome. Margin negative (R0) resection is the goal for the rhabdomyosarcoma. Given its aggressive histology and the possibility of incomplete resection, chemoradiation will likely be pursued. The CHOP induction chemotherapeutic regimen for Hodgkin’s lymphoma has some degree of overlap with the VAC protocol for rhabdomyosarcoma (vincristine being used for both). The challenge for the chemotherapist is the dose frequencies and potential toxicities that these patients may experience.

## Conclusions

Patients with Hodgkin’s lymphoma that present with a second primary tumor usually do so as a complication of chemoradiation. Cancer screening is conducted regularly in HL patients receiving treatment. However, presenting with Hodgkin’s lymphoma and a second primary tumor simultaneously is exceedingly rare and poorly described in the literature. This can be a challenging diagnosis to make, and clinicians must be aware that such entities exist. When synchronous masses are present, or a new mass is discovered in a patient with Hodgkin’s lymphoma prior to treatment, one must be careful not to assume that they are the same entity. When in doubt, a tissue diagnosis should be pursued.

## Ethics approval and consent to participate

Not applicable.

## Consent for publication

Consent was obtained from the patient for publication of relevant medical information.

## Availability of data and materials

Not applicable.

## Abbreviations

CHOP: cyclophosphamide, doxorubicin, vincristine, and prednisone
HL: Hodgkin’s lymphoma
RMS: rhabdomyosarcoma
VAC: vincristine, dactinomycin, and cyclophosphamide
